# Effects of simulated litter inputs on ecological niches and interspecific connectivity of alpine meadow plants

**DOI:** 10.3389/fpls.2025.1525474

**Published:** 2025-02-20

**Authors:** Weishan Lin, Kejia De, Xuemei Xiang, Tingxu Feng, Fei Li, Xijie Wei

**Affiliations:** College of Animal Husbandry and Veterinary Science, Qinghai University, Xining, Qinghai, China

**Keywords:** litter, alpine meadows, ecological niche, interspecific connectivity, species diversity

## Abstract

**Introduction:**

Plant litter enters the soil as the main nutrient for alpine meadow ecosystems, and the amount of litter input has a significant effect on alpine meadow plant diversity, ecological niches, and interspecific connectivity.

**Methods:**

The ecological niche width, ecological niche overlap and interspecific associations of alpine meadow species in the Sanjiangyuan area of the Qinghai-Tibetan Plateau were investigated using ecological statistical methods, and the competitive linkages between species and limited resources within the community and the stable coexistence among populations under different levels of plant litter inputs were explored.

**Results:**

(1) Litter inputs could significantly increase the plant diversity and aboveground biomass of alpine meadows, and the species with the highest importance value was *Kobresia humilis* Clarke. (2) compared with the control, with the increase of litter inputs, the ecological niche widths of the major plant species and ecological niche overlap values were significantly increased. And the F2 treatment had 15 major species totaling 105 pairs, and there were 82 pairs of ecological niche overlap values ≥0.950, accounting for 78.1%. (3) The correlation analysis between species showed that the negative correlation between species decreased and then increased with the increase of litter input. There were 3 pairs with Ac ≥ 0.25 under F2.

**Discussion:**

A moderate amount of litter input (F2) is beneficial to the structural stability of alpine meadow ecosystems. Excessive litter inputs may break the original balance of alpine meadow ecosystems and affect plant growth strategies. This study lays a foundation for the conservation of vegetation diversity and long-term use of grassland resources in degraded alpine meadows on the Tibetan Plateau.

## 1 Introduction

Against the background of global climate change and increasing human activities, the structure and function of ecosystems are facing great challenges, especially the fragile alpine ecosystems. Alpine meadows in China are mainly distributed on the Qinghai-Tibetan Plateau and in the alpine belts of various high mountain systems, with a total area of about 8.7×10^9^ hm^2^, accounting for 22.1% of the national grassland area, which is one of the largest and most important types of grassland in China (Wang et al., 2018). And it plays a key role in maintaining biodiversity, regulating climate and promoting carbon cycle. In recent years, the alpine meadow ecosystem in the Sanjiangyuan area has been seriously degraded due to global warming and intensified human activities such as grazing (Tang et al., 2014). The study showed that with the increase of degradation, the species composition of alpine meadows decreased with the increase of degradation, and the yield and quality of grassland as well as the vegetation cover and biodiversity gradually decreased (Zhang et al., 2019), which changed the nutrient return status of the litter (Li et al., 2014). As a key hub of the plant-soil-atmosphere material cycle, plant litter matter is the main source of soil nutrients, and changes in the quality and quantity of litter matter in alpine meadows will lead to changes in the structure of above-ground plant communities (Yang et al., 2020). With the unique geographic location and high altitude of the Sanjiangyuan area, the alpine meadow ecosystem is extremely sensitive to external disturbances, especially the dynamics of plant litter, which may directly affect the relationship between plant community structure and ecological niche function.

Since the concept of ecological niche was proposed by Grinnell in 1917, it has attracted the attention of many ecologists both at home and abroad, and has been widely studied, which has led to the wide application of the theory in the aspects of plant interspecific relationships, biodiversity, community structure and succession, and population evolution (Peng and Wang, 2016). Ecological niches are the resources occupied by populations in time and space and their functional relationships and roles with related populations (Liu et al., 2019), and are important indicators of the status and role of species in the biome (Avolio et al., 2019). Ecological niches width and ecological niches overlap values are ecological niches description methods used by numerous scholars. Ecological niches width is the magnitude of adaptation of a species in its environment (Wu et al., 2022a). Ecological niche overlap values are ecological niches with temporal or spatial similarities between species, reflecting the phenomenon of two or more species living in the same space and time sharing or competing for resources together (Gu et al., 2019; Pastore et al., 2021). Niche characteristics (niche width and overlap) reflect a species’ ability to utilize resources and its role within the community, while interspecific association refers to the spatial co-occurrence of species, largely determined by their coexistence in a specific environment (Su et al., 2015). Therefore, the study of ecological niche characterization is essential for understanding coexistence mechanisms and predicting community succession. Interspecific relationships are complex networks of relationships formed by direct or indirect interactions between species in a community (Ma et al., 2021). Inter-specific association (inter-specific association) is a type of inter-species relationship, which refers to the spatial distribution of species that are unevenly distributed due to habitat differences, but are related to each other to a certain extent (Yue et al., 2017). The alleviation of resource limitations also increases above-ground biomass, shifting competition from nutrient acquisition to light capture, indirectly reducing niche overlap and breadth, thereby intensifying interspecific competition (Yan et al., 2020). Integrating niche characteristics with interspecies associations provides a more comprehensive and effective understanding of community composition, species interactions, and predictions of population dynamics in grassland ecosystems (Hu et al., 2022). Research has shown that as grassland ecosystems transition from low-stability pioneer communities to highly stable climax communities, positive correlations between species gradually increase (Löffler and Pape, 2020). Conversely, when grassland ecosystems are in an unstable state, negative correlations between species tend to rise (Cassini, 2020). Studies have indicated that in the later stages of artificial grassland vegetation recovery, species tend to occupy narrower niches, leading to stable communities and harmonious coexistence. Over time, the impact of interspecies associations on community stability increases, making it a valuable tool for quantifying community structure (Wu et al., 2022b).

The research of litter began with the study of forest litter and has mostly focused on the study of litter’s effect on increasing soil fertility (Wood et al., 2006), improvement of the soil environment (Cheng et al., 2006) and promotion of material cycles (Zhu et al., 2002). Existing studies have focused on qualitative research on the effects of litter on plant community structure and function in temperate grasslands and typical grasslands (Wen et al., 2003; Wang et al., 2013). As an important component of the material cycle in alpine meadow ecosystems, changes in the amount of litter inputs can affect plant species composition, community structure, and plant competition for resources and utilization strategies. Different intensities of grazing are common on the Tibetan Plateau, resulting in differences in the amount of litter material returned to the surface and entering the soil (Zou et al., 2016). Differences in the amount of litter material will change the soil supply of nutrients needed for plant growth and development, destroying the original measurement of plant resource utilization, increasing the ecological adaptability of plants to the environment, intensifying the competition for nutrients among plant species, and leading to changes in the ecological localization of plant species and interspecific correlations (Xiang et al., 2024). Nevertheless, there is still a relative paucity of research on how litter inputs affect plant ecological niches (i.e., the temporal and spatial positions occupied by species in the community and their relationships with the environment) and interspecific associations (the relationship between species interactions) in alpine meadows. Whether increased litter inputs will lead to reconfiguration of plant ecological niches and adjustment of interspecific relationships is unknown. Hence, exploring the response mechanisms of changes in plant community characteristics, ecological niche indices and interspecific associations under different levels of litter inputs is crucial for understanding the complex effects of different grazing intensities on alpine meadows plant communities as well as providing data and theoretical support for biodiversity conservation.

Therefore, in this study, the alpine meadow in Zhenqin Town, Yushu Tibetan Autonomous Prefecture, Qinghai Province was used as the research object, and the sealing treatment was used as the basis to simulate the effects of litter input on the plant material composition and community structure of alpine meadow in the Sanjiangyuan area, and the experiments of litter input at different levels (F0 (CK)), 20% of the standard amount (F1), 50% of the standard amount (F2) and 100% of the standard amount (F3) were set up to analyze the Plant community characteristics, ecological niche width, ecological niche overlap index and interspecific association index changes. Our objectives were to determine (1) What are the dynamic changes of plant community characteristics and ecological niche characteristics of alpine meadows under different levels of litter treatments? (2) To quantitatively assess the impacts of litter inputs on the interspecific associations of alpine meadow plants and their feedback mechanisms? This will be of great significance for the continuous performance of the ecosystem services of alpine meadows in the Sanjiangyuan area, and will help researchers engaged in this field to crystallize the scientific problems.

## 2 Materials and methods

### 2.1 General information about the study area

The experiment was carried out at the Sanjiangyuan Ecosystem Field Scientific Observatory of the Ministry of Education, which is located in Zhenqin Town, Chengduo County, Qinghai Province (latitude 33°24′30″N, longitude 97°18′00″E), with an altitude of 4270 m. The climate is typical of a continental plateau, and the annual average temperature ranges from -10.3°C to 4.6°C. The annual average precipitation is 614.1 mm, which is mainly distributed between June and September. This grassland is a moderately degraded alpine meadow (De, 2014), and the main grasses in the alpine meadow are *Elymus nutans* Griseb., *Poa annua* L., *Koeleria macrantha* L., *Stipa capillata* L., *Oxytropis ochrocephala* Bunge., *Taraxacum mongolicum* L*., Potentilla nivea* L., *Anemone cathayensis* Kitag., *Gentiana scabra* Bunge., *Thalictrum alpinum* L., *Kobresia humilis* Clarke., *Pedicularis* L., *Lithophytum comptum* L., and other plants. The soil is alpine meadow soil with pH 6.92, organic matter content of 2.36%, quick-acting nitrogen content of 14.0 mg/kg, quick-acting phosphorus content of 7.0 mg/kg and quick-acting potassium content of 76.5 mg/kg (Lin et al., 2024).

### 2.2 Research methodology

#### 2.2.1 Experimental design

Alpine meadows that were not grazed during the growing season were selected for the study during the vegetation rejuvenation period in early May 2024 in Zhenqin Township, Chengduo County. Alpine meadows with uniform plant growth and flat terrain were selected for the simulated litter input experiment. To ensure the smooth running of the experiment, the study area was protected by an electric fence around the test area. In this study, the experiment was carried out in selected sample plots within the enclosure, each plot being 1 m^2^(1 m×1 m) in size. In alpine meadow ecosystems, more than 90% of the net production of plants is returned to the surface in the form of litter (Loranger et al., 2002; Fanin and Bertrand, 2016). Due to the perennial low temperature in the Sanjiangyuan area, the decomposition process of litter is extremely long, so glucose (C_6_(H_2_O)_6_) was chosen to replace plant litter in the simulation experiment in this study. Based on the consideration of maintaining the ecosystem function of alpine meadows and the research results of alpine meadows in the Sanjiangyuan area (Xiao et al., 2021; Zhou et al., 2021). And the glucose was added according to the 2% APC (Above-ground community carbon content of vegetation) (Gao, 2022), and the selected amount of glucose added was 17.424 g/m^2^.

Therefore, four treatments were set up in this study, 20% of the standardized amount (F1), 50% of the standardized amount (F2), 100% of the standardized amount (F3) and F0 (CK) (**Table 1**). In the experiment, each experimental plot was separated by a 20-mm-thick polyethylene material in order to prevent the flow of the saccharose solution between the different plots (the depth of the partitions was about 60 cm). The glucose from each treatment was dissolved in 3L of tap water and shaken well to ensure that no solute remained in the beaker and spray bottle. After shaking well, the glucose solution was evenly sprayed on the surface of the test sample with a small spray bottle (Gao, 2022). And equal amount of tap water was sprayed in the control group (F0). The plots were 1×1 = 1m² with 1m interval between plots and 4 replications for a total of 4×4 = 16 plots.

**Table 1:** Experimental design for litter addition.

Treatment	C Additions	Glucose Levels	Quantity of water added	Plot size (m^2^)	Repetition number
F0(CK)	0 g N m²	0 g/m²	3L	1	4
F1	1.39 g N m²	3.48 g/m²	3L	1	4
F2	3.48 g N m²	8.71 g/m²	3L	1	4
F3	6.97 g C m²	17.42 g/m²	3L	1	4

#### 2.2.2 Sample collection

Plant community characterization was conducted in late July 2024 during the peak vegetation growth season on treatments set up in the simulated litter input testbed. The size of the sample frame area is 50 cm × 50 cm were randomly placed in each treatment, and all the species within the sample boxes were recorded, the cover of community species and species sub-cover were determined using the pin-prick method, the heights of 10 mature individuals were measured flush with the ground using a steel ruler (if the number of plant individuals did not reach 10 plants, then all measurements were needed to obtain the average value), and the frequency and multiplicity of community species were determined by the counting method. All the plants in the sample plot were cut off flush with the ground and placed in an envelope bag in a cool place. The above-ground parts of the plants were placed in an oven and dried at 80°C for 48 hours until a constant weight was reached, and the above-ground biomass of the plants (AGB) was measured.

### 2.3 Calculation method of indicators

(1) Species importance value

Importance value (IV) refers to the ecological importance of a species, which can represent the degree of dominance of a species in a community, and is calculated as the sum of relative height (RH), relative cover (RC), and relative biomass (RB). The mean value of RH, RC, and RB was calculated as follows (Tao, 2009):


$$IV=\frac{RH+RC+RB}{3}$$

Species Richness Index (R): R = S;

Shannon-Wiener Diversity Index (H): *H* ′ = − ∑ *P_i_ In Pi*;

Pielou Index (E): E=H/lnS

where H is the diversity index and S is the number of plant species.

(2) Ecological niche width and ecological niche overlap

The systematic sampling method was used to investigate the characteristics of plant communities, and the “Levins” method was used to calculate the ecological niche widths and ecological niche overlap values of dominant species with the following formulas (Yang et al., 2024):


$$B_{i}=-\sum_{j=1}^{r}*(P_{ij}\text{ln}P_{ij})$$

$$N_{0}=\sum_{j=1}^{r}*P_{ij}P_{kj}/\sqrt{{\left(\sum_{j=1}^{r}*P_{ij}\right)}^{2}{\left(\sum_{j=1}^{r}*P_{kj}\right)}^{2}}$$

in the formula: *r* is the number of resource states; *j* is the resource; *P_ij_* is the proportion of individuals of species *i* utilizing resource *j* to the number of all individuals of that species; *P_kj_* is the proportion of individuals of species *k* utilizing resource *j* to the number of all individuals.

(3)Inter-species pair connectivity

The magnitude of interspecific connectivity was measured by the interspecific connectivity coefficient (Ac) (Xu et al., 2016).


$$A_{c}=\frac{ad-bc}{\left(a+b)(b+d\right)},(ad\geq bc)$$

$$A_{c}=\frac{ad-bc}{\left(a+b)(a+c\right)},(bc>ad,d\geq a)$$

$$A_{c}=\frac{ad-bc}{\left(b+d)(d+c\right)},(bc>ad,d<a)$$

in the formula: the interval of *Ac* is [-1,1], when the value of *Ac* is closer to 1, it indicates stronger positive association, when the value of *Ac* is closer to -1, it indicates stronger negative association, and when the value of *Ac* is 0, it indicates that the species are independent of each other (Yin et al., 2023). When AC ≥ 0.67, there is a strong positive association; 0.67 > AC > 0 indicates a weak positive association, AC = 0 indicates no association, -0.67 < AC < 0 indicates a weak negative association, and AC ≤ -0.67 also indicates a strong negative association.

### 2.4 Statistical analysis of data

Microsoft Excel 2010 was used to preliminarily organize the sample survey data and calculate the species importance values. SPSS 26.0 software was used to One-way analysis of variance (ANOVA) and least significant difference (LSD) *post hoc* test were conducted to test the effects of different litter levels on plant species diversity index and biomass, which were significant at the 0.05 confidence level. The ggplots2 program package in R4.4.1 software was used to lollipop species ecological niche widths, the “Pheatmap” package was used to heat map ecological niche overlap values, and the “Spaa” package was used to calculate species ecological niche characteristics and interspecific connectivity.

## 3 Results and analysis

### 3.1 Community dominant species composition and species diversity

**Figure 1:**
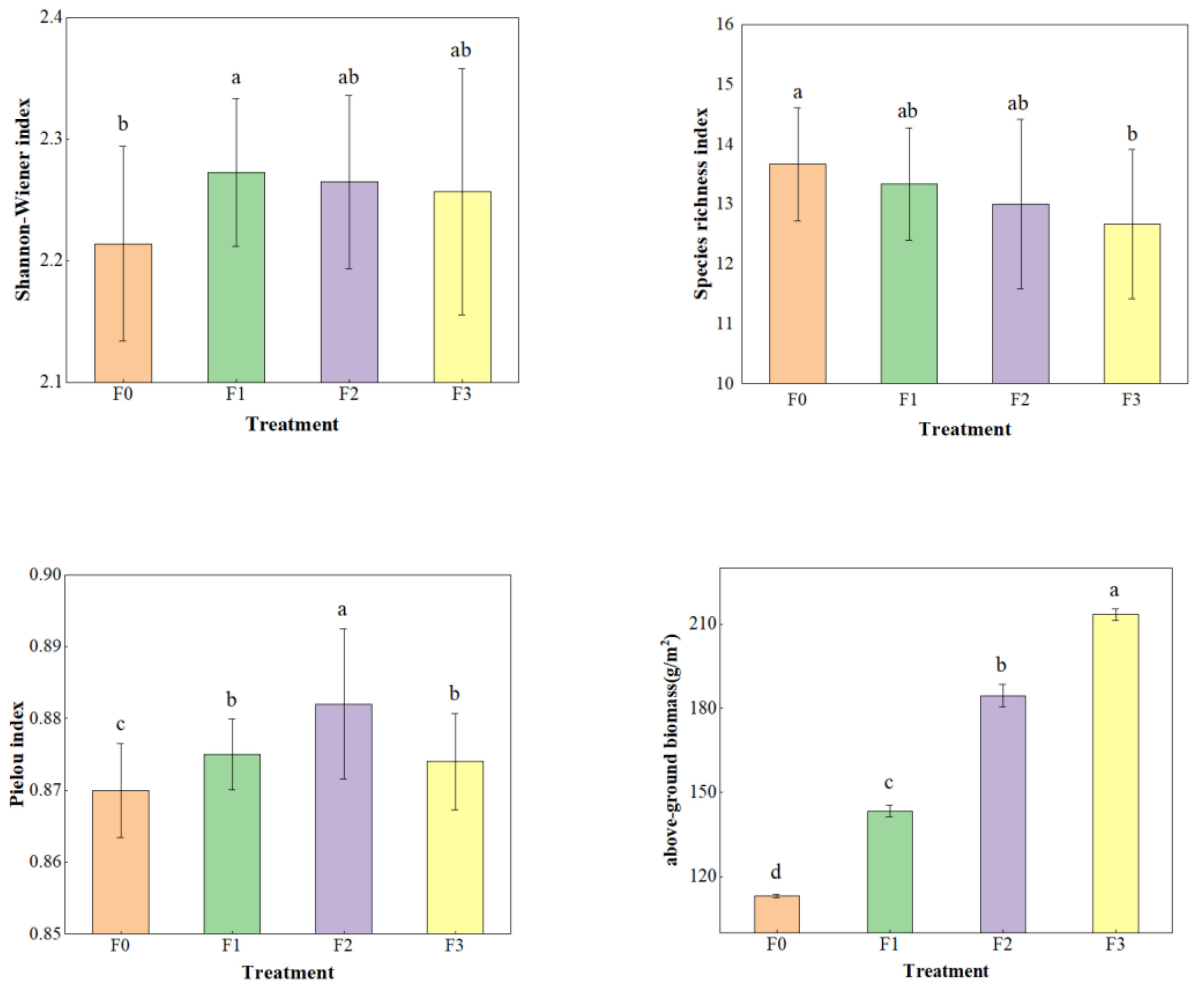
Species diversity and biomass under litter inputs. Lowercase letters in the figure replace differences between treatments (*P<0.05*).

As shown in **Table 2**, a total of 19 species of plants were found in this plant community characterization survey. Among them, there were 16 species in F0 and F3, and 15 species in F1 and F2. The importance values of plant species in alpine meadows under different levels of litter inputs varied, with the importance values of F0 and F1 being greater than 0.09 for *Elymus nutans*, *Oxytropis ochrocephala* and *Kobresia humilis*. Under F2 and F3 treatments, species importance values greater than 0.09 were found for *Elymus nutans* and *Kobresia humilis*. Litter inputs significantly increased plant diversity and aboveground biomass in alpine meadows in **Figure 1** (*P<0.05*). Shannon-Wiener index, Pielous index and aboveground biomass were significantly higher than that of F0 (*P<0.05*). Species richness index showed the opposite trend, and F0 was much larger than the other treatments (*P<0.05*).

**Table 2:** Main species and their abbreviations and importance values.

Serial No.	Spicious	Abbreviations	Important value(%)			
			F0	F1	F2	F3
1	*Elymus nutans* Griseb.	Ed	0.0996284	0.0944339	0.1081583	0.1135051
2	*Poa annua* L.	Pa	0.0643175	0.0547406	0.0703112	0.0700528
3	*Koeleria macrantha* L.	Km	0.0643577	0.0579275	0.0577338	0.0665577
4	*Stipa capillata* L.	Sc	0.0544728	0.047454	0.046616	0.0155251
5	*Oxytropis ochrocephala Bunge*.	Oo	0.0999001	0.0916514	0.085227	0.0896575
6	*Taraxacum mongolicum* L.	Tm	0.0453655	0.04014	0.039841	0.0382453
7	*Potentilla nivea* L.	Pc	0.0496179	0.072002	0.0809712	0.0795602
8	*Anemone cathayensis* Kitag.	Ac	0.0446693	0.0821021	0.0600829	0.0663
9	*Gentiana scabra Bunge*.	Gs	0.0323062	0.0383566	0.0358123	0.0388577
10	*Thalictrum alpinum* L.	Ta	0.028762	0.0308647	0.0310334	0.0325347
11	*Kobresia humilis* Clarke.	Kh	0.3221411	0.3205452	0.3168417	0.3088278
12	*Pedicularis* Linn.	Pe	0.0084929	0.0319497	0.0191872	0.0241894
13	*Lithophytum comptum* L.	Lt	0.0196482	0.0183561	0.0191652	0.019328
14	*Aster tataricus* L.	At	0	0.0137093	0	0
15	*Kobresia tibetica Maximowicz* L.	Kt	0	0.005767	0	0
16	*Anemone coronaria* L.	An	0.0201625	0	0.0196939	0
17	*Bistorta vivipara* (L.) Gray	Bv	0.0105998	0	0.009325	0.0097825
18	*Gentiana macrophylla* Pall.	Gm	0.0355581	0	0	0.0142053
19	*Ranunculus japonicus* Thunb.	Rj	0	0	0	0.0128707

### 3.2 Effects of litter input on ecological niche width in alpine meadows

**Figure 2:**
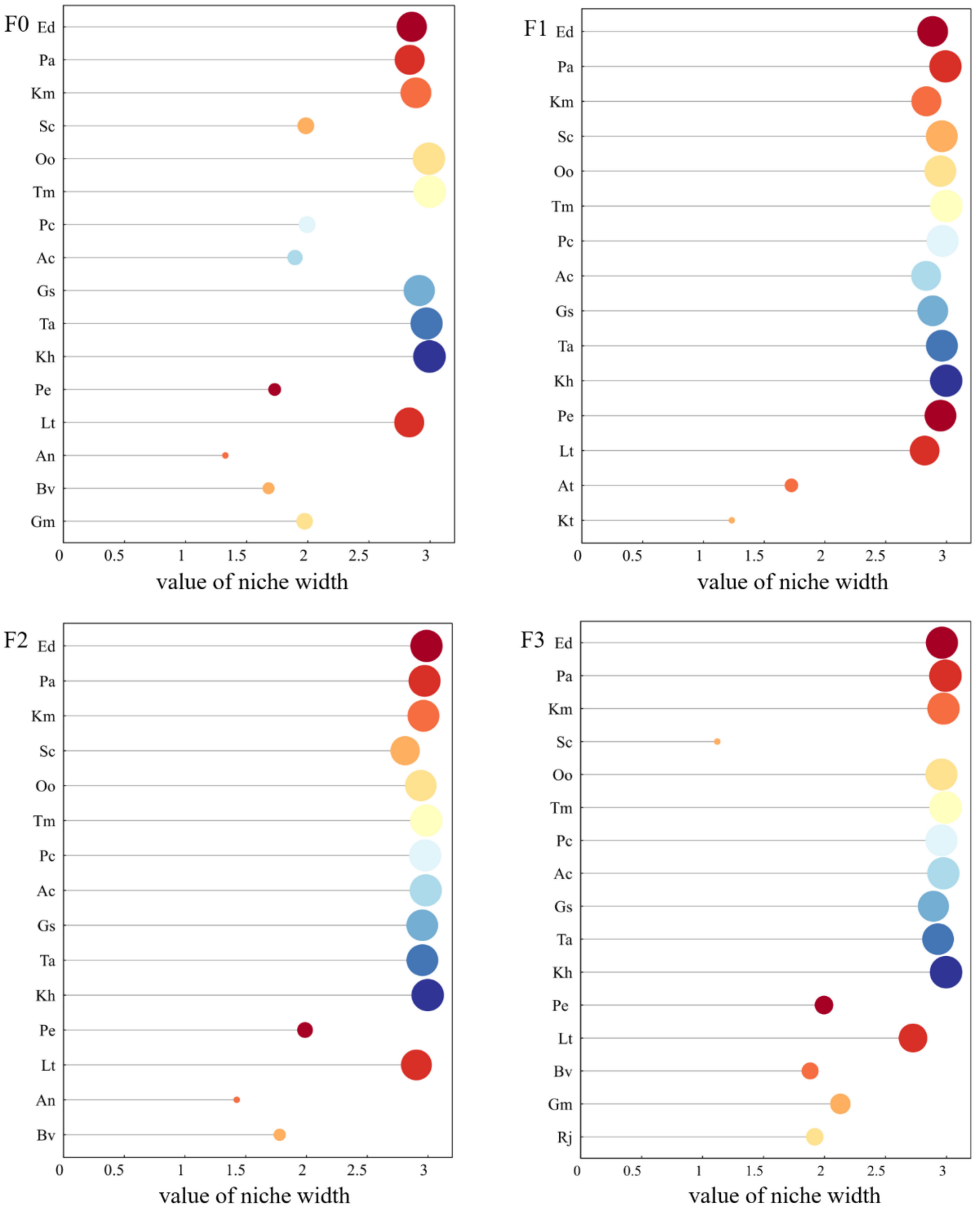
Changes in the ecological niche width of major species under litter inputs. Abbreviated names of plant species are provided in **Table 1**.

Ten plants with high significant values were selected in each sample plot for ecological niche and interspecific association analysis. As can be seen from **Figure 2**, the ecological niche widths of the major species in the study area changed with the different levels of litter input. At F0, the ecological niche widths of the major species with larger values were *Taraxacum mongolicum* (2.997), *Kobresia humilis* (2.995), *Oxytropis ochrocephala* (2.990), and *Thalictrum alpinum* (2.971). Ecological niche widths were greater for litter inputs of F1 for *Poa annua* (2.991), *Taraxacum mongolicum* (2.999), and *Kobresia humilis* (2.997). At F2, the ecological niche widths of the main species were larger for *Elymus nutans* (2.988), *Poa annua* (2.972), *Taraxacum mongolicum* (2.987) and *Kobresia humilis* (2.998). At F3, the major species with larger ecological niche widths were *Poa annua* (2.992), *Taraxacum mongolicum* (2.993), *Anemone cathayensis* (2.975) and *Kobresia humilis* (2.997).

### 3.3 Effects of litter inputs on ecological niche overlap in alpine meadows

**Figure 3:**
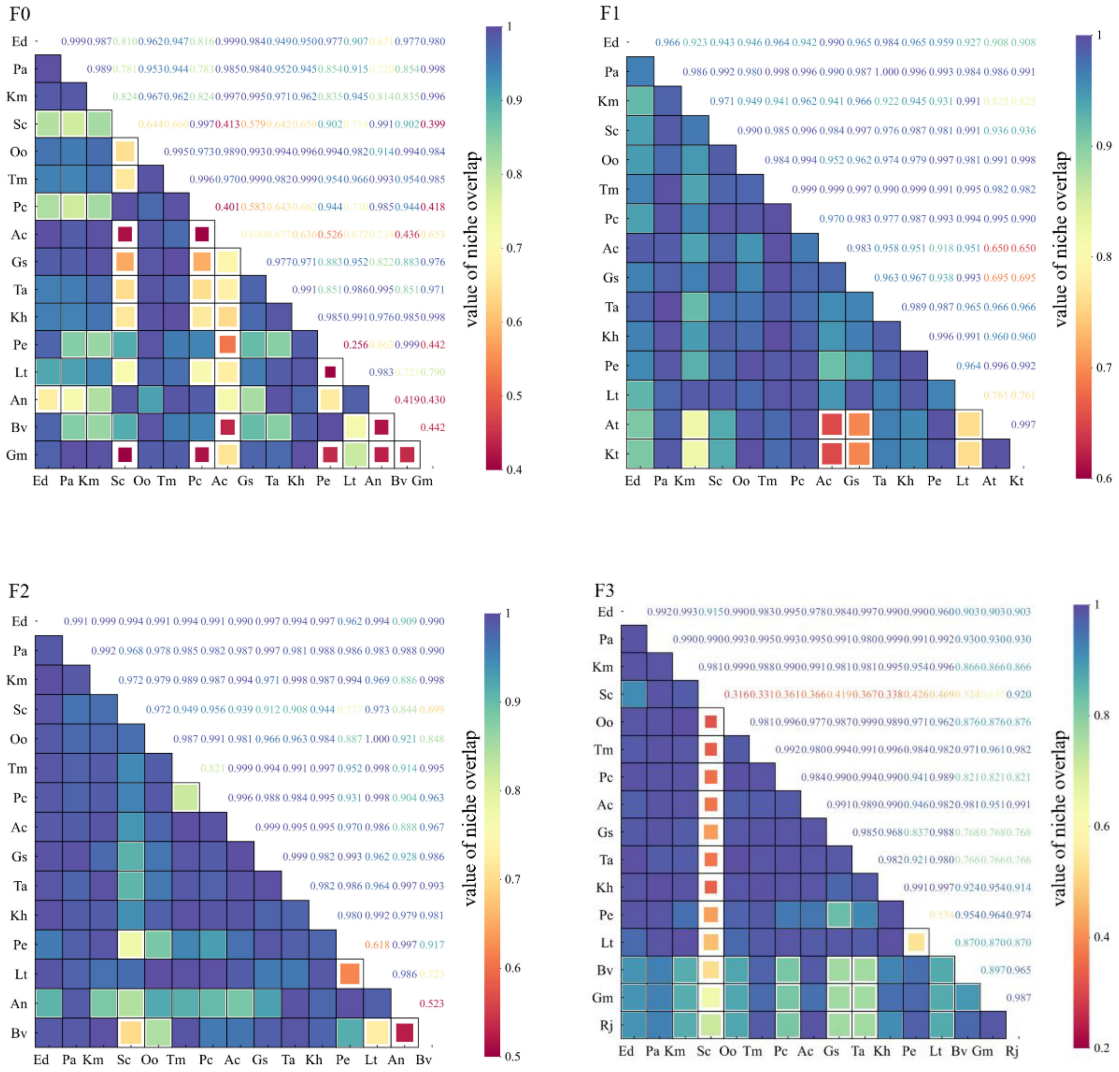
Changes in ecological niche overlap of major species under litter inputs.

The ecological niche overlap values of the main species of alpine meadow plants under different levels of litter inputs were significantly different (**Figure 3**). In F0, there were 120 pairs of 16 species, 62 pairs (51.67%) of ecological niche overlap values ≥0.950; 10 pairs (8.33%) of 0.95~0.90; 38 pairs (31.67%) of 0.90~0.50; and 10 pairs (8.3%) of ≤0.5. In F1, there were 105 pairs of 15 species, 81 pairs (77.14%) of ≥0.950; 16 pairs (15.3%) of 0.90~0.95. In F2, 15 species totaling 105 pairs, with 82 pairs (78.1%) of ecological niche overlap values ≥0.950, 12 pairs (11.43%) of 0.90-0.95, and 11 pairs (10.48%) of ≤0.90. In F3, 16 species totaling 120 pairs, with 10 pairs (8.3%) of ecological niche overlap values ≥0.950; with 76 pairs (63.33%) of ≥0.950; 11 pairs (9.47%) of 0.90~0.95; 24 pairs (20%) of 0.899~0.50; and 9 pairs (7.5%) of <0.5.

### 3.4 Effects of litter inputs on interspecific connectivity of alpine meadow plants

**Figure 4:**
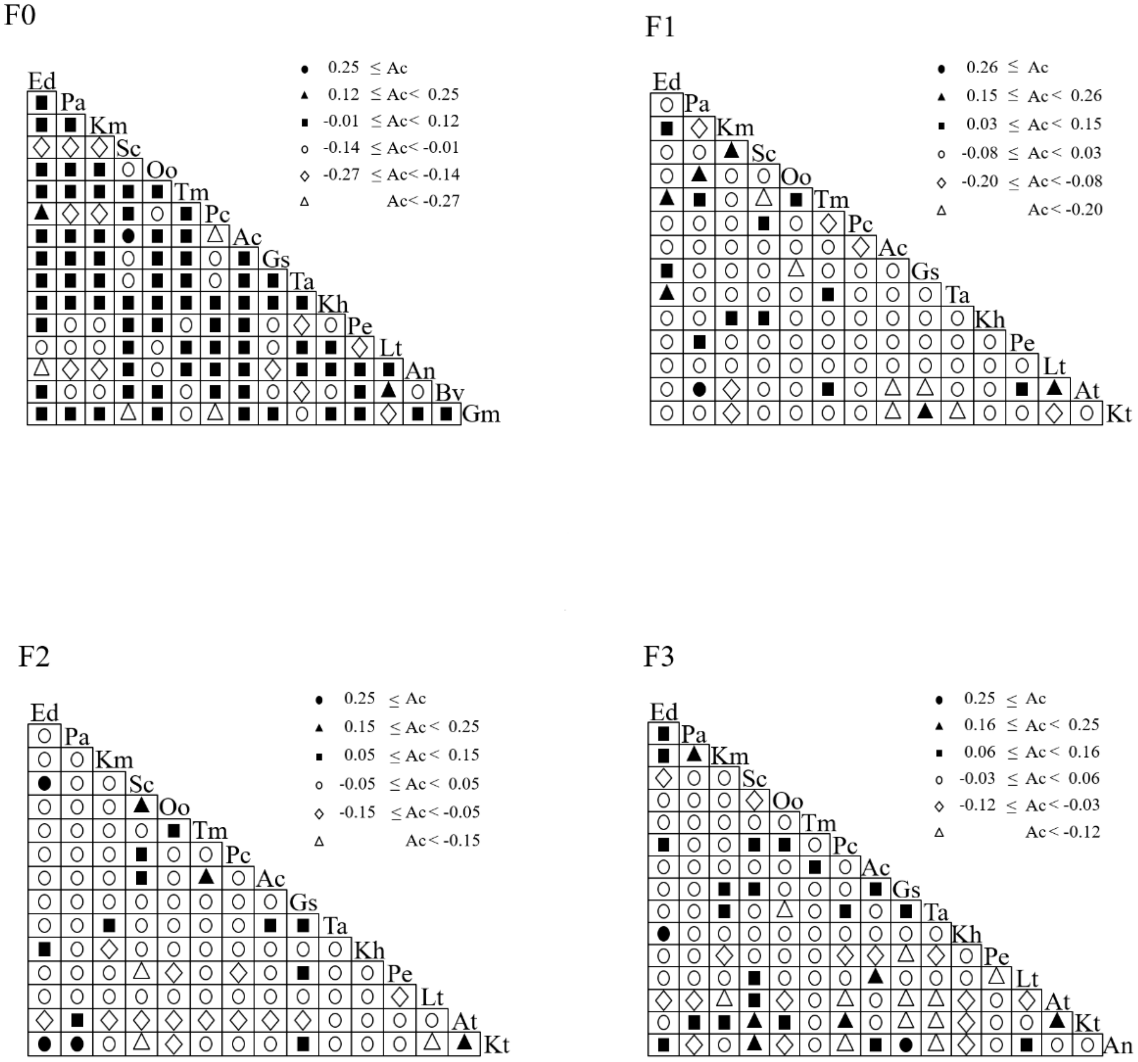
Ac index of major species under litter inputs.

The above analysis discussed the effects of different levels of litter input on plant species diversity, biomass, ecological niche width and overlap in alpine meadows, and in order to further reflect the degree of likelihood of the co-occurrence of different species, the interspecific associations of the different species in the spatial distribution were characterized by calculating the interspecific associations. As can be seen from **Figure 4**, different levels of litter input had different effects on the interspecific association coefficient Ac index. In F0, there was one pair with Ac ≥ 0.25; two pairs with 0.12 ≤ Ac < 0.25; 77 pairs with -0.01 ≤ Ac < 0.12; 25 pairs with -0.14 ≤ Ac < -0.01; 11 pairs with -0.27 ≤ Ac < -0.14; and four pairs with Ac < - 0.27. In F1, there was 1 pair with Ac ≥ 0.26; 6 pairs with 0.15 ≤ Ac < 0.26; 11 pairs with 0.03 ≤ Ac < 0.15; 75 pairs with -0.08 ≤ Ac < 0.03; 6 pairs with -0.20 ≤ Ac < -0.08 and 6 pairs with Ac < -0.20. In F2, there were 3 pairs with Ac ≥ 0.25; 3 pairs with 0.15 ≤ Ac < 0.25; 10 pairs with 0.05 ≤ Ac < 0.15; 73 pairs with -0.05 ≤ Ac < 0.05; 13 pairs with -0.15 ≤ Ac < -0.05, and 3 pairs with Ac < -0.15. In F3, there were 2 pairs with Ac ≥ 0.25; 6 pairs with 0.16 ≤ Ac < 0.25; 20 pairs with 0.06 ≤ Ac < 0.16; 66 pairs with -0.03 ≤ Ac < 0.06; 15 pairs with -0.12 ≤ Ac < -0.03 and 11 pairs with Ac < -0.15.

**Figure 5:**
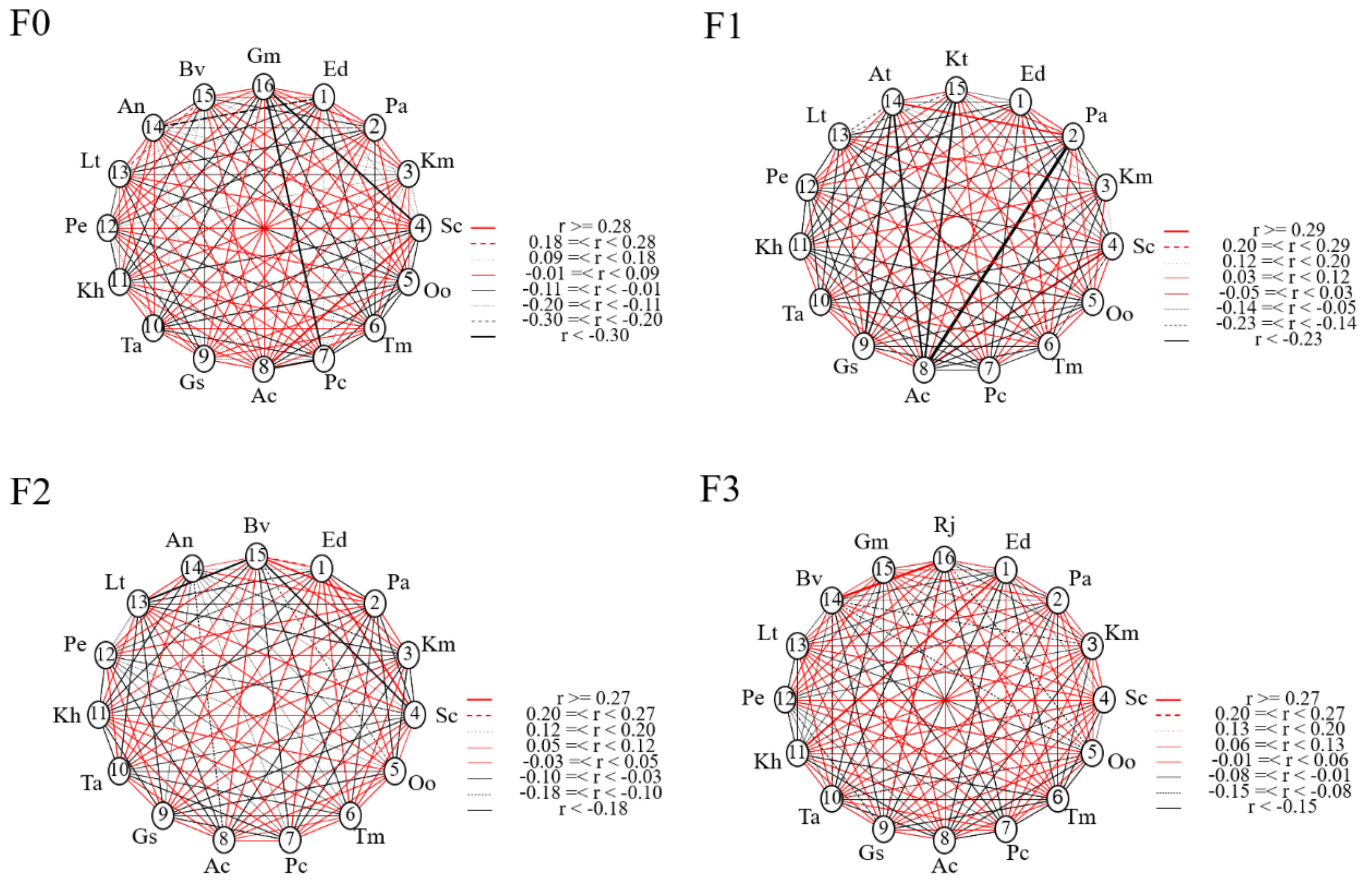
Network linkage map of Ac species under litter inputs.

Based on the results of the Ac test, the following figure shows the effect of different levels of litter inputs on the inter-species associations of plant species (**Figure 5**). In F0, one pair of species showed significant positive correlation, *Stipa capillata* with *Anemone cathayensis*. And two pairs of species showed highly significant negative correlation, *Gentiana macrophylla* with *Stipa capillata* and *Potentilla nivea*. In F1, one pair of species showed significant positive correlation as *Poa annua* with *Aster tataricus* and one pair of species showed significant negative correlation as *Poa annua* with *Anemone cathayensis*. In F2, two pairs of species showed significant positive correlation, *Elymus nutans* with *Stipa capillata* and *Poa annua* with *Bistorta vivipara*; two pairs of species showed significant negative correlation, *Bistorta vivipara* with *Lithophytum comptum* and *Stipa capillata*. In F3, two pairs of species showed a significant positive association, *Elymus nutans* with *Kobresia humilis* and *Bistorta vivipara* with *Ranunculus japonicus.* The rest of the species were not associated. The Ac network linkage map was generally consistent with the Ac index results, with most species generally not significantly correlated or independently distributed (i.e., when the Ac value was close to 0), and a few species showing mutualistic or competitive relationships when the demand for environmental resources was high.

## 4 Discussion

### 4.1 Major species diversity

The plant species diversity index is an effective indicator for assessing plant community heterogeneity and successional processes (Wei et al., 2024). It has been found that plant litter accumulation affects the soil physical environment, nutrient cycling and light availability, influences species competition (Meier and Bowman, 2010) and regulates species diversity (Dong et al., 2018). In alpine meadows on the Tibetan Plateau, litter accumulation inhibited the growth of miscellaneous grass plants and altered community species competition, resulting in species loss or changes in the composition of plant functional groups, which led to a decrease in plant community species richness and dominance with increasing amounts of litter, but did not affect the Shannon diversity index (Zou et al., 2016). Degradation of alpine meadows in the Sanjiangyuan area poses a serious threat to the normal fulfillment of ecosystem services in alpine meadows (Sun et al., 2012).The results of this study showed that different levels of litter input significantly increased the plant diversity and aboveground biomass of alpine meadows, and the Shannon-Wiener index, Pielous index, and aboveground biomass were significantly higher than that of F0 (*P<0.05*), which is inconsistent with the findings of existing studies (Wang et al., 2010; Qin et al., 2024). This may be due to the degradation of alpine meadows in the Sanjiangyuan area, in which the dominant forage grasses such as *Gramineae* and *Salviaceae* were gradually replaced by miscellaneous grasses, the structure of plant communities changed, and the diversity of species declined. Plant litter regulates the establishment of dominant species and seedlings of new species in alpine meadows, and increases species richness and Shannon’s diversity index (Wang et al., 2010). Plant litter cover affects seed germination and seedling establishment by changing light and potential survival substrates (Xia et al., 2021), thus affecting species diversity.

Another explanation is that soil acts as a donor of plant nutrients, supplying them during the growing season. It may be because the study area belongs to a moderately degraded alpine meadow, and the increase in the amount of organic carbon entering the soil led to the initiation of the excitation effect of the soil carbon pool and the intensification of soil microbial activity, which altered the microbial community structure and life strategies (Che et al., 2020). With the increase in microbial activity, the expression of functional genes drives soil microorganisms to decompose complex organic matter (e.g., plant residues, animal excreta, etc.) into simple organic and inorganic nutrients that can be easily absorbed by plants through the secretion of various enzymes and other biologically active substances. The nutrient elements released during decomposition are further transformed by soil microorganisms to form forms that are easily absorbed and utilized by plants. The rich source of nutrients promotes the growth and development of plants, allowing some of the more diminutive species to be supplied with nutrients and increasing species diversity. In summary, the increase in litter input showed positive effects on both plant species diversity and biomass, further reflecting that grazing in degraded meadows on the Tibetan Plateau is not conducive to grassland development, and that new management practices should be developed subsequently.

There are also some negative effects of litter inputs on alpine meadow ecosystems. For example, the input of litter may break the existing nutrient balance of alpine meadow ecosystem, and the input of liter may reduce the nitrogen supply capacity of the soil in a short period of time, which may aggravate the degradation of the soil (Jiang et al., 2013). This is mainly due to the fact that a large amount of organic matter will be released during the decomposition of litter material, which may compete with the inorganic nitrogen in the soil at the early stage of decomposition, leading to a decrease in the inorganic nitrogen (especially nitrate nitrogen) content in the soil. Litter matter can increase soil water-holding capacity and soil water content by absorbing and intercepting water, reducing surface evaporation, and improving soil structure (Li et al., 2000; Pan et al., 2004). Increased soil moisture may also lead to reduced soil aeration, which modulates the respiration pattern and nutrient uptake mechanisms of plant roots. Certain chemicals produced during the decomposition of plant litter are related to the inhibitory effects on microorganisms, and interspecific interactions such as nutrient transfer and chemical inhibition may also occur (Li et al., 2016). However, the exact mechanism of this effect still needs further research. Therefore, the amount of litter inputs should be reasonably added in grassland management to fully utilize its positive effects and avoid potential negative impacts. Further studies on the response mechanisms of alpine meadows with different degradation levels to litter inputs will be beneficial to the adaptive management of alpine meadows.

### 4.2 Ecological niche characterization and interspecific connectivity

Niche width reflects a species’ adaptability to environmental conditions and its capacity to utilize ecological resources (Fajardo and Siefert, 2019). A larger niche width indicates a greater ability to exploit resources (Yang et al., 2021). Some scholars have found that the size of ecological niche overlap values of alpine meadow plant species is not related to the ecological niche allocation mechanism (Zhang et al., 2020a, b). Species with large ecological niche widths were usually considered to have higher resource utilization capacity and high ecological niche overlap with other species (Cuesta et al., 2020). This is consistent with the results of this study. For example, in the present study, it was found that the largest ecological niche widths under F1 and F2 treatments were *Taraxacum mongolicum* (2.999) and *Kobresia humilis* (2.997), respectively, and both of them had a high degree of ecological niche overlap (>0.94) with other species. Possible reasons are that alpine meadows on the Tibetan Plateau are built up with cold-tolerant and cold-adapted mesic perennial herbaceous plants, with dense grass clusters and low grass layers (Hao et al., 2020); and the structure of the large number of irregularly piled up “grass felt layers” formed by long years of low temperatures is not easy to be altered (Shi et al., 2015). During the degradation of alpine meadows, graminoid species gradually declined, and miscellaneous grasses heavily encroached on the survival space. For example, the survival space of *Elymus nutans*, *Poa annua*, *Koeleria macrantha*, *Stipa capillata* was compressed. As the main dominant species in alpine meadows, *Kobresia humilis* has a strong ecological adaptability with a short but complex root system and strong nutrient absorption and utilization ability.

In conditions of resource scarcity, niche overlap reflects both the ecological similarity between species and the level of competition among them. Conversely, under resource-rich conditions, niche overlap merely indicates ecological similarity or the occupation of similar ecological space by species (Wang et al., 2023). Some studies have found that ecological niche overlap among species is also related to species characteristics, and some species with larger ecological niche widths may exhibit different values of ecological niche overlap due to factors such as resource allocation strategies (Schellenberger Costa et al., 2018). Our results showed that *Elymus nutans* (2.988), *Taraxacum mongolicum* (2.987) and *Potentilla nivea* (2.977) had greater ecological niche widths at a litter input of F2. Nevertheless, the trend of overlap of staggered bands was higher in *Potentilla nivea* and *Elymus nutans* (0.99), while the trend of overlap of ecological niches was reduced in *Potentilla nivea* and *Taraxacum mongolicum* (0.82). Litter inputs enhanced plant productivity and canopy height, shifting competition among plant species from below-ground nutrient competition to above-ground competition for light (Xiang et al., 2024). This shift means that taller or faster-growing species can capture more light per unit area compared to shorter species, intensifying competitive exclusion (Niu et al., 2018).

Interspecific relationships have long been a cornerstone of community ecology and are essential for understanding species distribution, community assembly, and responses to environmental changes (Ding and Ma, 2021). Interspecific associations can be used as an indicator of species ecological niche overlap (Banerji, 2021). Some studies have shown that the higher the degree of positive interspecific linkages, the greater the overlap of ecological niches (Zhang et al., 2020a). Nonetheless, the degree of positive interspecific associations does not reflect the degree of ecological niche overlap in all resource dimensions, and the reasons for negative interspecific associations are more complex, with lower values of ecological niche overlap due to habitat differences and higher values of ecological niche overlap due to environmental resource competition (Gu et al., 2019). Our results showed that the negative correlation between species decreased and then increased with the increase of litter input, and the mean value of the overlap of ecotone bands between species decreased and then increased, suggesting that the mechanism of interactions between populations was unfavorable to one or two species. The negative correlation reflected the adaptation and response of different species in alpine meadows to different litter inputs. Possibly when because the addition of moderate amounts of litter material leads to differences in nutrient use, growth rates, and life cycles of plant species, resulting in greater ecological niche differentiation and allowing more species to coexist in the same habitat (Tian et al., 2022). Nonetheless, over-addition of litter additions may increase the nutrient uptake capacity of certain plants, leading to increased interspecific competition. This competition is a key factor in ecological niche characterization, as dominant species compete for available nutrients, inhibiting the growth of smaller species and reducing the ecological niche dimension (Farrer and Suding, 2016).

Due to the regional characteristics of alpine, sensitivity, and vulnerability (Chen et al., 2014), the alpine meadows on the Tibetan Plateau are sensitive to increased impacts of climate change, environmental factors, and human activities (Jiang et al., 2017). Some studies have shown that increased nitrogen deposition in the Sanjiangyuan area has led to an increase in litter nitrogen content (Vestgarden, 2001). This study did not explore how the regulatory mechanism of litter inputs to alpine meadows is affected by nitrogen deposition. Therefore, follow-up studies should explore the regulatory mechanisms of litter inputs on species community construction and species competition in alpine meadows in the context of increasing nitrogen deposition and global warming on the Tibetan Plateau. Follow-up studies should utilize Grass Watch 1.0 platform, ArcGIS and Global Mapper software in combination with meteorological information for long-term monitoring of alpine meadows, exploring the interspecific relationships between species in alpine meadow communities, and the ecological relationships between interdependent and mutually limiting plant species, which will help to understand the competition mechanism of alpine meadow plant communities for resources and the strategy of nutrient utilization. The study will help to understand the competition mechanism of alpine meadow plant community for resources and the strategy of nutrient utilization.

## 5 Conclusion

Litter input significantly increased plant diversity, aboveground biomass, and species ecological niche width in alpine meadows, and the species with the highest importance value was *Kobresia humilis* Clarke. Under F2, there were 82 pairs of 15 species with ecological niche overlap value ≥0.950 out of 105 pairs, accounting for 78.1%. The correlation analysis among species showed that the negative correlation among species first decreased and then increased with the increase of litter input, and there were 3 pairs of Ac ≥0.25 under F2 treatment. This indicates that a moderate amount of litter input is beneficial to the structural stability of alpine meadow ecosystems, while too much litter input will break the original equilibrium of degraded alpine meadows and aggravate species competition. These findings provide key data and theoretical insights for understanding the competitive mechanisms and changes in plant community diversity in degraded alpine meadows. Furthermore, exploring the role of other environmental factors such as soil pH, soil temperature and moisture in alpine meadows in shaping plant community responses to global climate change. This research will contribute to the development of biodiversity conservation strategies and sustainable management practices for alpine meadows on the Tibetan Plateau.

## Data Availability

The original contributions presented in the study are included in the article/**Supplementary Material**. Further inquiries can be directed to the corresponding author.
